# Characterization of methanol utilization negative *Pichia pastoris* for secreted protein production: New cultivation strategies for current and future applications

**DOI:** 10.1002/bit.27303

**Published:** 2020-02-24

**Authors:** Domen Zavec, Brigitte Gasser, Diethard Mattanovich

**Affiliations:** ^1^ Department of Biotechnology University of Natural Resources and Life Sciences Vienna Austria; ^2^ CD‐Laboratory for Growth‐Decoupled Protein Production in Yeast, Department of Biotechnology University of Natural Resources and Life Sciences Vienna Austria

**Keywords:** bioprocess, growth decoupled, *Komagataella phaffii*, methanol induction, Mut^–^

## Abstract

The methanol utilization (Mut) phenotype in the yeast *Pichia pastoris* (syn. *Komagataella* spp.) is defined by the deletion of the genes *AOX1* and *AOX2*. The Mut^−^ phenotype cannot grow on methanol as a single carbon source. We assessed the Mut^−^ phenotype for secreted recombinant protein production. The methanol inducible *AOX1* promoter (P_*AOX1*_) was active in the Mut^−^ phenotype and showed adequate eGFP fluorescence levels and protein yields (Y_P/X_) in small‐scale screenings. Different bioreactor cultivation scenarios with methanol excess concentrations were tested using P_*AOX1*_HSA and P_*AOX1*_vHH expression constructs. Scenario B comprising a glucose‐methanol phase and a 72‐hr‐long methanol only phase was the best performing, producing 531 mg/L HSA and 1631 mg/L vHH. 61% of the HSA was produced in the methanol only phase where no biomass growth was observed, representing a special case of growth independent production. By using the Mut^−^ phenotype, the oxygen demand, heat output, and specific methanol uptake (*q*_methanol_) in the methanol phase were reduced by more than 80% compared with the Mut^S^ phenotype. The highlighted improved process parameters coupled with growth independent protein production are overlooked benefits of the Mut^−^ strain for current and future applications in the field of recombinant protein production.

## Graphical abstract

Two key genes necessary for methylotrophy in the yeast *Pichia pastoris* were disrupted. The strain was characterized in terms of bioprocess parameters and recombinant protein production under methanol induction. Different bioprocesses were tested and compared to the commonly used Mut^S^ strain and bioprocess. The strain showed reduced heat evolution and oxygen demand as well as the capability of recombinant protein production without a noticeable biomass increase.

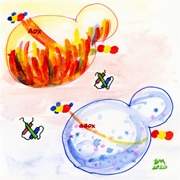

## 1 INTRODUCTION

*Pichia pastoris* (syn. *Komagataella* spp.) is a well‐known protein production host in the biopharmaceutical and industrial fields and is capable of the appropriate folding and secretion of eukaryotic recombinant proteins (Mattanovich et al., 2012). Frequently, the methylotrophic properties of *P. pastoris* are exploited to induce, control, and produce recombinant proteins in high quantities. Methylotrophy in *P. pastoris* is dependent on two alcohol oxidases, *AOX1* and *AOX2*, necessary for the first step of the methanol metabolism. Deletion of the more abundantly expressed *AOX1* gene leads to the methanol utilization slow (Mut^S^) phenotype (Cregg et al., 1987; Cregg, Madden, Barringer, Thill, & Stillman, 1989). *P. pastoris* Mut^S^ strains are commonly used for recombinant protein production under methanol induction because of the lower growth rate and methanol uptake rate (Krainer et al., 2012; Looser et al., 2015). Protein production in these strains is achieved by the induction of methanol inducible promoters, as for example, the *AOX1* promoter (Cregg et al., 1987). For this purpose, a limiting methanol only feed or a methanol co‐feed with glucose or glycerol is required to induce gene expression at the desired time in the bioreactor cultivation process (Cos, Ramón, Montesinos, & Valero, 2006; Looser et al., 2015; Potvin, Ahmad, & Zhang, 2012). However, methanol uptake by the Mut^S^ strains still poses disadvantages. The methanol metabolism is rather inefficient as the first step of methanol oxidation by alcohol oxidase requires a direct electron transfer from methanol to oxygen and therefore increases the oxygen demand and generates excessive heat as well as oxidative stress (Couderc & Baratti, 1980). Compared with other microbial expression systems, this is the main constraint to consider when upscaling a *P. pastoris* process (Krainer et al., 2012; Theron, Berrios, Delvigne, & Fickers, 2018). Deletion of the *AOX1* and *AOX2* genes obstructs the methanol metabolism, effectively creating a methanol utilization negative phenotype (Mut^−^) which is not able to grow on methanol as a sole carbon and energy source (Cregg et al., 1989; Sreekrishna et al., 1989). The use of a methanol metabolism deficient strain would, on the other hand, solve the above‐mentioned disadvantages. This led us to further investigate if the Mut^−^ strain is able to produce secreted proteins when induced with methanol. We further investigated whether an additional carbon and energy source is necessary for protein production (Chiruvolu, Cregg, & Meagher, 1997).

## 2 MATERIALS AND METHODS

### 2.1 Generation of Mut^S^ and Mut^−^ strains

The strains *P. pastoris* (syn. *Komagataella phaffii*) CBS2612 Mut^S^ and Mut^–^ were created by deleting first the *AOX1* gene and subsequently the *AOX2* gene including parts of their promoter regions 567 bp and 503 bp upstream of the ATG by the split marker approach (Gasser et al., 2013; Heiss, Maurer, Hahn, Mattanovich, & Gasser, 2013). The split marker cassette was carrying the antibiotic resistance cassette for geneticin flanked by LoxP sites. For selection, YPD with 500 µg/ml geneticin was used. The antibiotic marker was later removed by the transformation of a Cre recombinase carrying plasmid (pTAC_Cre_Hph_Mx4; Marx, Mattanovich, & Sauer, 2008). The knockouts were verified by the polymerase chain reaction (PCR) amplification over the *AOX1* and *AOX2* locus and subsequent sequencing of PCR amplicons.

### 2.2 Generation of protein production strains

eGFP was used to screen for the *AOX1* promoter expression levels. The linearized vector (*Asc*I) BB3aN_pAOX1_eGFP_CycTT was transformed into the *AOX1* terminator (Prielhofer et al., 2017) and selected on YPD plates with 100 ug/mL nourseothricin. For secreted protein production, the Mut^S^ and Mut^−^ strains were transformed with the pPM2pN21_pAOX1_HSAopt_CycTT vector (Mut^−^ P_*AOX1*_HSA & Mut^S^ P_*AOX1*_HSA), carrying codon‐optimized human serum albumin (HSA) with the native secretion sequence (Puxbaum, Gasser, & Mattanovich, 2016) and the pPM2pZ30_pAOX1_αMF‐vHH_CycTT vector (Mut^−^ P_*AOX1*_vHH and Mut^S^ P_*AOX1*_vHH) carrying a codon‐optimized variable region of a camelid antibody (vHH) with *a Saccharomyces cerevisiae* α‐mating‐type secretion sequence. Vector generation and transformation have been described in detail elsewhere (Gasser et al., 2013; Stadlmayr et al., 2010). Both vectors were linearized with XmnI for integration into the *PGI1* locus. Transformants were selected on YPD plates with 100 µg/mL nourseothricin or 25 µg/mL zeocin, respectively.

### 2.3 P_*AOX1*_ expression level screening

The P_*AOX1*_eGFP reporter construct allowed for determination of P_*AOX1*_ activity by measuring the fluorescence by flow cytometry (Ata, Prielhofer, Gasser, Mattanovich, & Çalık, 2017; Hohenblum, Borth, & Mattanovich, 2003; Prielhofer et al., 2013; Stadlmayr et al., 2010). Ten Mut^S^ and Mut^−^ BB3aN_pAOX1_eGFP_CycTT transformants were picked for screening. The clones were inoculated in duplicates at a start OD_600_ of 1 into 24 deep well plates with 2.5 ml of ASMv6 minimal media (6.3 g/L (NH_4_)_2_HPO_4_, 0.8 g/L (NH_4_)_2_SO_4_, 0.49 g/L MgSO_4_*7H_2_O, 2.64 g/L KCl, 0.054 g/L CaCL_2_*2H_2_O, 22 g/L citric acid monohydrate, 1.47 ml/L PTM1 trace metals, 20 ml/L NH_4_OH (25%); pH set to 6.5 with KOH) with 25 g/L polysaccharide and 0.3% amylase (m2p‐labs GmbH) to achieve glucose limiting conditions. Two hours later, the cultures were induced with either 0.2% or 1% (vol/vol) methanol. eGFP fluorescence was measured 4 and 20 hr after induction using a flow cytometer (Beckman Coulter, Inc.). The FX values were calculated (FX = (FL1/FS^1,5^) × 8,000) normalizing the fluorescence signal to the cell size (Hohenblum et al., 2003).

### 2.4 Screening of secreted protein production

Overnight precultures in liquid YPD with 100 µg/mL nourseothricin or 25 µg/mL zeocin were washed and inoculated into 24 deep well plates with a starting OD_600_ of 8 and 2 ml of ASMv6 minimal media. Because of the different physiological needs of the Mut^S^ and Mut^−^ strains, we compared different screening protocols varying in methanol induction time points and the total amount of methanol used (Table 1). Twelve and six millimeters slow glucose release feedbeads (Kuhner Shaker GmbH) with a 48 hr average release rate of 0.60 and 0.21 mg/hr per feed bead were used to supply the cultures with a limiting amount of glucose (Jeude et al., 2006). At the end of the cultivation phase, 1 ml of the screening culture was harvested by centrifugation at 16,000 g for 10 min. The supernatant was stored at − 20°C for later analysis, the pellet was used for the determination of the wet cell weight (WCW). Twelve transformants were screened per expression construct. The transformants with no detectable protein or with nearly double the concentration of the population average were removed from the data as outliers before further analysis. These outliers are likely to be double copy clones that would impact the comparability or did not incorporate the expression construct at all (Aw & Polizzi, 2013; Schwarzhans et al., 2016).

**Table 1:** Overview of tested screening protocols for secreted protein production

Protocol	Duration, hr	Feedbeads, mm	Start OD_600_	Methanol shot	Total methanol, vol/vol	Methanol shot time points, hr
Standard	48	12	8	4 ×	3.5%	4^a^, 19, 27, 43
One shot	48	3 × 6	8	1 ×	1%	3
Two shot	48	3 × 6	8	2 ×	2%	3, 23
One shot – extended	72	3×6	8	1 ×	1%	3
Two shot – extended	72	3×6	8	2 ×	2%	3, 43

^a^ First methanol shot was only 0.5% (vol/vol).

### 2.5 Gene copy number determination

Gene copy number (GCN) was determined by quantitative PCR (qPCR), as already described (Prielhofer et al., 2013). Briefly, genomic DNA was isolated with a Wizard® Genomic DNA Purification Kit (Promega Corp.). The GCN was determined by the relative quantification of the P_*AOX1*_ sequence compared with CBS2612 WT carrying a single copy of the P_*AOX1*_ sequence and the CBS2612 Mut^−^ as the negative control having no P_*AOX1*_ sequence and normalized to the house‐keeping gene *ACT1*. The amplifications were carried out using the Biozym Blue SʹGreen qPCR mix (Biozym Scientific GmbH) and the RT‐PCR cycler Rotor‐Gene Q (QIAGEN GmbH). Primer sequences are found in Table S1.

### 2.6 HPLC methanol determination

The methanol concentrations in the bioreactors were determined by HPLC (Shimadzu Corp.) with an Aminex HPX‐87H (Bio‐Rad Laboratories, Inc.) column using 4 mM H_2_SO_4_ as mobile phase at 0.6 ml/hr at 60°C. The detector was a RID‐10A at 40°C (Shimadzu Corp.; Pflügl, Marx, Mattanovich, & Sauer, 2012).

### 2.7 Bioreactor cultivations

The bioreactor cultivations were done in a DASGIP® Parallel Bioreactor System (Eppendorf AG). The Batch media with a starting volume of 300 ml consisted of BSM with 40 g/L glycerol as a carbon source (Mellitzer et al., 2014). The feed medium consisted of 50% (wt/wt) glucose or 60% (wt/wt) glycerol and PTM1, the methanol feed was diluted to 50% (vol/vol). All strains were cultivated in two parallel biological replicates. The pH was set to 5.5 and controlled through DASGIP® control software by either 25% NH_4_OH in Scenarios A, D, and B for vHH production or 12.5% NH_4_OH in Scenarios B and C for HSA production and by 10% phosphoric acid by manual addition. The feed rates of glucose and glycerol and in some cases that of methanol were monitored gravimetrically and controlled using a custom script.

### 2.8 Bioreactor cultivations, Scenario A: the constant glucose‐methanol co‐feed strategy

**Figure 1:**
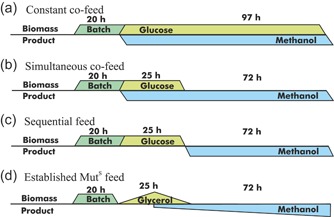
Schematic representation of the bioreactor cultivation strategies used. (a) Constant glucose‐methanol co‐feed. (b) Simultaneous glucose‐methanol co‐feed and methanol only feed. (c) Sequential glucose and methanol only feed. (d) Established Mut^S^ protocol [Color figure can be viewed at http://wileyonlinelibrary.com]

After the batch phase on glycerol for approximately 20 to 21 hr, a constant 50% glucose feed was started at 2.4 ml/hr corresponding to 1.45 g/hr of pure glucose. Methanol was added to the cultivation media to a target concentration of 1.5% (vol/vol) (Figure 1a). The methanol concentration was checked daily by HPLC and kept in excess at 0.5–1.5% (vol/vol) by a methanol feed.

### 2.9 Bioreactor cultivations, Scenarios B and C: simultaneous and sequential induction strategy with a methanol only feed phase

The CBS2612 Mut^−^ strains were cultivated in three phases. (a) Phase one was a batch phase on glycerol for approximately 20 to 21 hr, followed by (b) phase two with a glucose feed at 4.8 ml/hr, corresponding to 2.9 g/hr of pure glucose for 25 hr. Induction of protein production was started by a methanol pulse to 1.5% (vol/vol) methanol. The methanol pulse was applied either at the start of phase two in Scenario B (Figure 1b) or at the start of phase three in Scenario C (Figure 1c). After the methanol addition, the concentration was checked at line with HPLC. The methanol input consisted of (a) a methanol feed and (b) daily methanol pulses to counteract methanol evaporation, dilution due to the glucose feed and potential oxidation. Therefore, the concentration was measured daily and a compensatory methanol pulse applied when the concentration was below the desired 1.5% (vol/vol). (c) Phase three was a methanol only feed phase with no additional glucose feed, the concentration was kept at 0.5–1.5% (vol/vol; Figure 1b,c). The dissolved oxygen (DO) control with a set point of 20% was disabled in this phase for the Mut^−^ cultivation and the aeration and mixing was set to 9.5 sL/hr and 750 rpm to avoid unnecessary swings and drops in DO.

### 2.10 Bioreactor cultivations, Scenario D: the Mut^S^ glycerol‐methanol co‐feed strategy

Scenario D was applied for the Mut^S^ strains as a comparison (Cos et al., 2006; Potvin et al., 2012). It was divided into four phases (Figure 1d). (a) Phase one consisted of the same BSM batch media with a glycerol batch phase for approximately 20 to 21 hr. (b) Phase two was a linearly increasing (*y* = 0.225x + 1.95) 60% glycerol feed for 8 hours followed by (c) phase three, an 18 hr co‐feed of 60% glycerol and 100% methanol. In the co‐feed, the 60% glycerol feed was linearly decreasing (*y* = 3.75 – 0.111x) and the methanol was linearly increasing (*y* = 0.028x+ 0.6). (d) Phase four was the methanol only feed phase with a linearly increasing methanol feed (*y* = 0.028x+ 1.10). Phase three of Scenario D and phase two of Scenario B represent the transition phase, phase four of Scenario D and phase three of Scenarios B and C correspond to the methanol production phase.

### 2.11 Bioreactor sampling

For Bioreactor sampling, the port was first flushed, then a 9 ml sample was taken and aliquoted into three preweighed 2 ml tubes. The aliquots were centrifuged for 10 min at 16,000 g at 4°C. The supernatants were processed directly for HPLC or frozen at −20°C. The pellets were dried at 105°C for at least 24 hr to determine the dry cell mass, with an OD_600_/DCW ratio of 5.0. If sampling just for HPLC analysis was necessary, only 2 ml of the sample was taken.

### 2.12 Determination of secreted proteins

The secreted proteins in the sample supernatant of the bioreactor cultivations and small‐scale screenings were analyzed in duplicates with the LabChip® GXII Touch™ (Perkin Elmer, Inc.) according to the manufacturerʹs recommendations (Prielhofer et al., 2018). The specific productivity between two time points *t*_0_ and *t*_1_ was calculated according to Equation (1) and the average overall *q_P_* according to Equation (2). *c_P_* is the product concentration in the supernatant and *F* = .0033 l·g^−1^ is the correction factor of the fraction of culture volume occupied by cells.

(1) $$q_{P}=\frac{\left(c_{P}\times (1-{\text{CDW}}_{1}\times F\right)}{\left(\frac{{\text{CDW}}_{0}+{\text{CDW}}_{1}}{2}\right)\times {∆t}_{0\to 1}}$$

(2) $$q_{P}=\frac{\left(\text{total}\;\text{protein}\right)}{\left(\text{average}\;\text{total}\;\text{CDW}\right)\times ∆t}$$

### 2.13 Cell viability measurements

Viability was measured using propidium iodide staining and flow cytometry (Beckman Coulter, Inc.). The bioreactor samples were diluted with PBS to an OD_600_ below 0.5 and mixed with 20 μM propidium iodide PBS solution in equal volumes (Hohenblum et al., 2003).

### 2.14 Estimation of the heat output

We estimated the heat of reaction based on oxygen transfer rates reported by the DASGIP® control software. The consumption of 1 mol O_2_ corresponds to the release of 460 kJ of heat (Cooney, Wang, & Mateles, 1969). As a control, we compared the data to the combustion energy of the consumed methanol fed into the reactors. The amount of oxidized methanol was calculated by the subtraction of the methanol fixed by the growing biomass based on the carbon to biomass ratio of 0.444 (Carnicer et al., 2009) and the remaining methanol in the medium at the end of the cultivation.

## 3 RESULTS

### 3.1 P_*AOX1*_ activity in Mut^−^ strains

**Figure 2:**
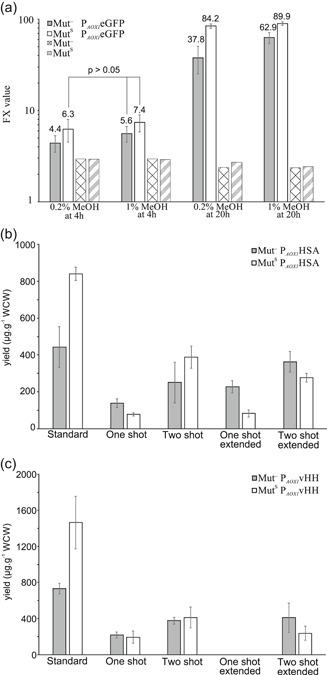
(a) Mean promoter expression levels with P_*AOX1*_eGFP of ten transformants, FX = (FL1/FS^1.5^) × 8,000. All measurements in pairwise comparison are significantly different according to Student's *t* test except for those indicated by *p* > .05. (b) Secreted protein production screening using different protocols with P_*AOX1*_HSA and (c) P_*AOX1*_vHH

We compared the P_*AOX1*_ expression levels of the Mut^S^ and Mut^−^ at 4 and 20 hr after induction with different methanol concentrations (Figure 2a). The differences in the mean values of the ten transformants are all statistically significant (Student's *t* test, *p* < .05) unless otherwise indicated on the figure. Twenty hours postinduction, the expression levels increased substantially compared with 4 hours. There was a 55% difference in expression levels between the Mut^S^ and Mut^−^ strains at 0.2% (vol/vol) methanol (FX value of 84.2 and 37.8). One percent (vol/vol) methanol led to a substantial increase in FX values for the Mut^−^strain and only a marginal increase for the Mut^S^ strain, the difference being only 30% (FX value of 62.9 and 89.9). This shows that the P_*AOX1*_ is suitable for protein production in the Mut^−^ strain and that compared with the Mut^S^ strain, the promoter activity drops at lower methanol concentrations.

### 3.2 Protein production screening of the Mut^−^ P_*AOX1*_HSA strain

The screening done according to Table 1 demonstrates that the Mut^−^ strain can produce secreted proteins with similar yields (Y_P/X_) as the Mut^S^ strain (Figure 2b). The highest yield for the Mut^−^ strain was achieved with the “Standard” protocol. In this case, the Mut^S^ still had a 1.9‐fold higher yield compared with the Mut^–^ strain. The Mut^−^ was outperforming the Mut^S^ in three out of the four adapted screening protocols where the total methanol content was lower. In terms of yield, the “Two shot – extended” strategy was only 18% behind than the “Standard” protocol for the Mut^−^ strain, but had higher absolute protein titers at 20.2 ± 3.4 µg/mL compared with 12.1 ± 3.5 µg/mL (“Standard” protocol). This makes protein concentration determination more accurate and thus better at discriminating differences between the tested transformants.

### 3.3 Protein production screening of the Mut^−^ P_*AOX1*_vHH strain

vHH was produced with higher yields than HSA (Figure 2c). Generally, the relative differences between the Mut^−^ and Mut^S^ are very similar to the HSA screening discussed before and follow the same pattern. The best performing protocol in terms of yield was again the established “Standard” protocol. The Mut^−^ had a 50% lower yield compared with the Mut^S^. The “Two shot – extended” protocol was the second best for the Mut^−^ strain and showed a clear advantage over the Mut^S^ strain. Concurrently, protein titers were again the highest with the “Two shot – extended” protocol at 23.5 ± 10.5 µg/mL compared with 14.9 ± 1.1 µg/mL (“Standard” protocol) confirming the result from the HSA screening. The protein titer in the “one shot – extended” protocol was below the limit of detection (Figure S1). We concluded that the “Two shot – extended” strategy was the best alternative screening protocol tested.

### 3.4 Selection of strains for bioreactor cultivation

An average performing clone from the screened strains was selected for bioreactor cultivation. The strains were confirmed by qPCR to carry a single copy of the integrated expression construct.

### 3.5 Bioreactor cultivation, Scenario A: a direct strain comparison of Mut^−^ and Mut^S^

**Figure 3:**
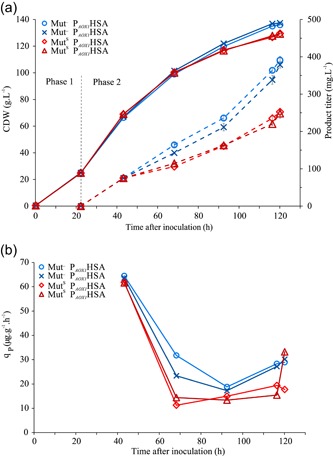
(a) Bioreactor cultivation of the Mut^−^P_*AOX1*_HSA and Mut^S^ P_*AOX1*_HSA with the constant co‐feed Scenario A. Full line is the CDW, the dotted line is the product titer. (b) Specific productivity over time. The Mut^−^ parallels are depicted in dark and light blue and the Mut^S^ in dark and light red [Color figure can be viewed at http://wileyonlinelibrary.com]

We checked the Mut^–^ secreted protein production of HSA during a constant glucose feed modeled after the conventional P_GAP_ process (Boer, Teeri, & Koivula, 2000; Looser et al., 2015) and additional methanol excess conditions for induction of P_*AOX1*_HSA (Figure 1a). The goal of the cultivation was to achieve growth controlled by glucose and to induce the *AOX1* promoter by methanol excess concentration. A feed rate of 2.4 ml/hr was selected so that an end CDW of 140 g/L was reached. For direct comparison, we cultivated the Mut^S^ P_*AOX1*_HSA strain alongside the Mut^−^ P_*AOX1*_HSA strain under the same conditions with equal amounts of methanol. After the batch phase indicated by a DO peak, a 50% glucose feed was started and a 1.5% (vol/vol) methanol pulse was applied. The methanol concentration of the two parallel reactors with the Mut^−^ P_*AOX1*_HSA strain was kept between 1.19% (vol/vol) to 1.5% (vol/vol) methanol (Table S2). The Mut^S^ P_*AOX1*_HSA strain was fed with an equal amount of methanol as the Mut^−^ P_*AOX1*_HSA strain. Both strains received a total of 25.2 ± 0.2 g of methanol. The methanol in the Mut^S^ P_*AOX1*_HSA cultivations was immediately consumed and was therefore at 0% (vol/vol). From the data presented (Figure 3), we confirmed that the induction of P_*AOX1*_ in the Mut^−^ strain is sufficient for secreted protein production and the Mut^−^ P_*AOX1*_HSA had a clear advantage with a 54% higher protein concentration over the Mut^S^ P_*AOX1*_HSA. The *q_P_* decreased over time (Figure 3b), this can be attributed to the decreasing growth rate caused by the constant glucose feed applied. Interestingly, the CDW of the Mut^S^ P_*AOX1*_HSA was lower by 5.6% and the viability by 1.4% points.

### 3.6 Bioreactor cultivation, Scenarios B and C: Mut^−^ process with a methanol only feed phase

**Figure 4:**
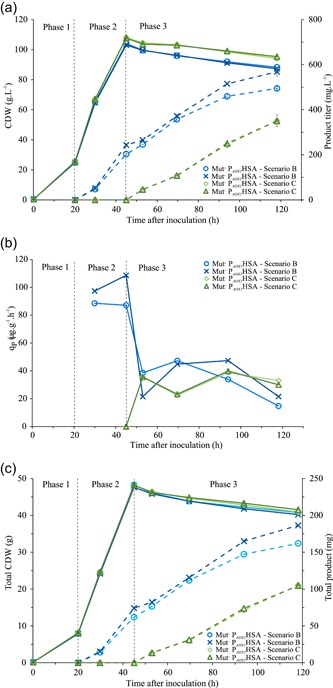
(a) Bioreactor cultivation of the Mut^−^ P_*AOX1*_HSA with Scenarios B and C. Full line is the CDW, the dotted line is the product titer. (b) Specific productivity over time. (c) Total CDW and protein in the cultivation vessel over time. The parallels are depicted in dark and light blue for Scenario B and in dark and light green for Scenario C [Color figure can be viewed at http://wileyonlinelibrary.com]

**Figure 5:**
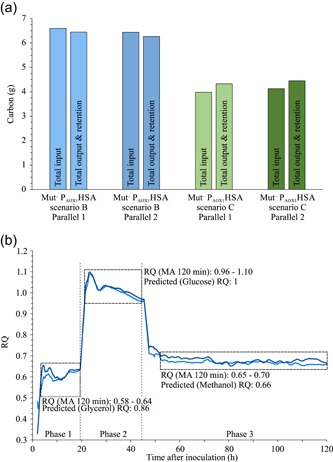
(a) Total carbon balance of all parallel cultivations. The parallels are depicted in dark and light blue or dark and light green, respectively. (b) 120 min moving average of the respiratory quotient for Scenario B [Color figure can be viewed at http://wileyonlinelibrary.com]

We tested if the Mut^–^ P_*AOX1*_HSA strain can produce secreted proteins with a methanol only feed without any additional carbon source. Therefore, the strains were cultivated in two different Scenarios B and C. The batch phase finished after approximately 20 hr, as indicated by a DO peak, a glucose feed was started for 25 hr. We induced the protein production with a methanol pulse to 1.5% (vol/vol) at the start of the glucose feed (Scenario B) or at the end of the glucose feed (Scenario C). The methanol concentrations were kept from 0.54% to 1.58% (vol/vol; Table S2). The secreted protein concentration in Scenario B reached 223.5 mg/L HSA by the end of the glucose feed and increased to 541 mg/L till the end of the methanol only feed phase. One hundred six milligrams of total HSA were produced in the methanol only feed phase corresponding to 61% of the total secreted protein produced with Scenario B. In Scenario C, the production was initiated with no glucose feed overlap. The concentration of the produced HSA was 351 mg/L, this accounted for 105 mg total protein produced, which is matching the produced amount in the methanol only feed phase of Scenario B (Figure 4a). This is additionally illustrated with the *q_P_* of Scenarios B and C. The average *q_P_* of 34.0 μg·g^−1^·hr^−1^ in the methanol only feed phase of Scenario B was similar to Scenario C with a *q_P_* of 32.9 μg·g^−1^·hr^−1^ (Figure 4b). However, the overall average *q_P_* of Scenario B was higher at 45.8 μg·g^−1^·hr^−1^ as production started already in the glucose co‐feed phase with a much higher *q_P_*. The difference in CDW between the Scenarios B and C is mostly due to dilution effects by the methanol feed. The calculated total biomass values were therefore similar for Scenarios B and C (Figure 4c). The secreted protein purity data of Scenario B were at 85% and for Scenario C at 79%. The cell viability in all reactors was very high (>99.51%). A total carbon balance of Phase 3, Scenarios B and C was calculated based on the methanol depletion in the methanol only feed phase, CO_2_ output, and an estimated evaporation rate of 50 mg·L^−1^·hr^−1^ based on experimental data (Figure 5a and Table S3). The RQ values in the methanol feed phase, Scenario B (Figure 5b) follow the predicted RQ for methanol oxidation.

### 3.7 Specific methanol uptake rate and methanol evaporation

We determined the specific methanol uptake rate (*q*_methanol_) of the biomass in Scenarios B and C using the average biomass concentrations over the entire methanol only feed phase. In this phase, the biomass concentration was stable, the methanol input was measured gravimetrically, and the residual methanol in the cultivation media at the end of the cultivation was subtracted. On average, a methanol uptake rate of 4.6 mg·g^−1^·hr^−1^ for Scenario B and 3.9 mg·g^−1^·hr^−1^ for Scenario C was calculated. To assess the amount of the *q*_methanol_ that can be accounted for by the evaporation of methanol out of the reactor, an evaporation control experiment without cells was made. Five hundred and three hundred ten milliliters of sterile batch media without glycerol were spiked with 1% (vol/vol) methanol and agitated at 750 rpm with a gassing rate at 9.5 sL/hr as would be the case in the methanol only feed phase of our process. Samples were taken at 3.5, 6.5, 22.5, 31, 47 hr and measured by HPLC. The methanol concentration in the bioreactor decreased linearly at a rate of 22 mg·L^−1^·hr^−1^ with 500 ml fill volume and 63 mg·L^−1^·hr^−1^ with 310 ml fill volume. Based on these results, we calculated that approximately 14% of the *q*_methanol_ can be explained by the evaporation of methanol from the cultivation media.

### 3.8 Bioreactor cultivation, Scenario B: production of a camelid vHH

**Figure 6:**
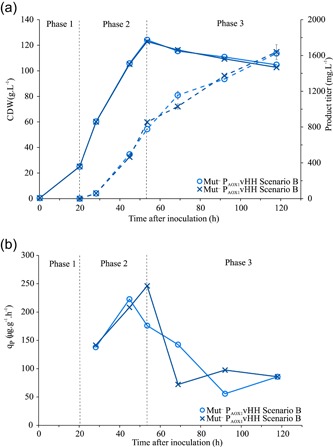
(a) Bioreactor cultivation results for the Mut^−^P_*AOX1*_vHHv with Scenario B. Full line is the CDW, the dotted line is the product titer. (b) Specific productivity over time [Color figure can be viewed at http://wileyonlinelibrary.com]

As the simultaneous induction protocol of Scenario B was the most productive feed strategy tested for the Mut^−^ P_*AOX1*_HSA strain, we decided to test and confirm the feeding strategy with the Mut^−^P_*AOX1*_vHH. To achieve higher biomass, the 4.8 ml/hr glucose feed was prolonged to 34 hr. As stated previously, protein production was induced by the addition of methanol to 1.5% (vol/vol) and was kept between 0.99% and 1.43% throughout the induction phase (Table S2). Due to the longer glucose feed phase, we achieved a higher CDW. The product formation, as well as the *q_p_* pattern, followed that of the Mut^−^ P_*AOX1*_HSA bioreactor cultivations, Scenario B (Figure 6a). A high *q_P_* in the glucose‐methanol co‐feed phase that reaches up to 216 μg·g^−1^·hr^−1^ was followed by a decrease in the methanol only feed phase (Figure 6b). The average productivity in the methanol feed phase was 88 μg·g^−1^·hr^−1^. This experiment confirms our findings with HSA and shows that the Mut^−^ strain under methanol induction can also be used to express secreted proteins in the >1 g/L range. A complete overview of all cultivations is available in Table S4.

### 3.9 A process comparison: Mut^−^ strain in Scenario B and Mut^S^ strain in Scenario D

**Figure 7:**
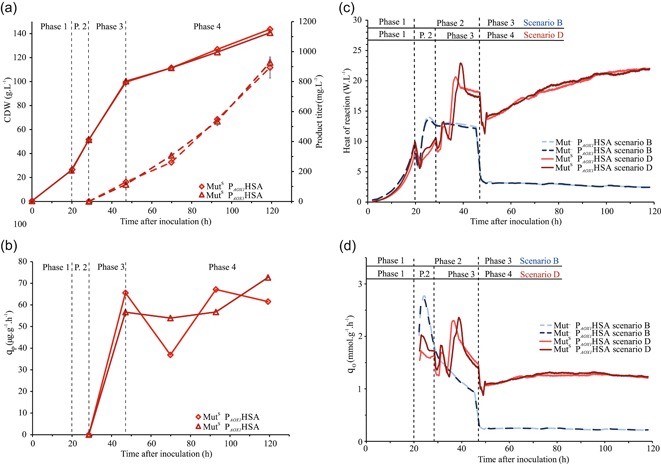
(a) Bioreactor cultivation results for the Mut^S^ P_*AOX1*_HSA with Scenario D. Full line is the CDW, the dotted line is the product titer. (b) Specific productivity over time. (c) Comparison of the heat of reaction per volume. (d) Comparison of specific oxygen uptake rate. The light and dark blue dotted line represent the Mut^−^ P_*AOX1*_HSA in Scenario B and the light and dark red full line the Mut^S^ P_*AOX1*_HSA in Scenario D [Color figure can be viewed at http://wileyonlinelibrary.com]

We cultivated the Mut^S^ P_*AOX1*_HSA with an established feeding strategy for the Mut^S^ strain (Cos et al., 2006; Potvin et al., 2012) and compared it with the Mut^–^ strain cultivated with strategy B. The Mut^S^ P_*AOX1*_HSA had reached 72% higher HSA concentration at the end of the cultivation, corresponding to a higher *q_P_* in the methanol feed phase by 82% at 62 μg·g^−1^·hr^−1^. However, the amount of methanol needed to sustain induction of the Mut^S^ P_*AOX1*_HSA is comparatively high. A total of 150.6 g of methanol was fed into the cultivation vessels, corresponding to six times the amount needed in the Mut^−^P_*AOX1*_HSA cultivation. During the methanol only feed phase, the Mut^S^ P_*AOX1*_HSA strain fixed approximately 53 g of methanol into the biomass and facilitated a biomass increase of 42.2%. In comparison, the biomass concentration of Mut^−^P_*AOX1*_HSA in the same phase remained stable. Approximately, 98 g of the methanol consumed by the Mut^S^ P_*AOX1*_HSA strain is oxidized and adds to the heat output and increases the oxygen uptake rate (Figure 7). Therefore, a big difference in oxygen demand and heat output can be observed between the two strains. The OTR needed to sustain the Mut^−^ strain in aerobic conditions during the methanol only feed phase is lower by 85%, from 149 to 23 mM/hr. We calculated the total heat output of the two cultivations and show that the reduced methanol uptake of the Mut^−^ P_*AOX1*_HSA strain accounts for an 84% reduction in heat output (Table 2). The viability at the end of the cultivation was lower for the Mut^S^ P_*AOX1*_HSA at 96.2% as well, most probably due to the stress added by the active methanol metabolism (Xiao, Zhou, Zhou, & Zhang, 2006).

**Table 2:** Comparison of key bioreactor cultivation parameters overall and in the methanol only feed phases of the Mut^−^P_*AOX1*_HSA strain in Scenario B and the Mut^S^ P_*AOX1*_HSA Scenario D

		Mut^−^ P_*AOX1*_HSA	Mut^S^ P_*AOX1*_HSA	
		Scenario B	Scenario D	Change
Overall	HSA concentration, mg/L	531	911	−42%
	*q_P,_* μg·g^−1^·hr^−1^	46	62	−26%
Methanol only feed phase: Phase 3 Scenario B, Phase 4 Scenario D	Heat production rate, W/L	3	19.2	−84%
	OTR, mM/h	23	149	−85%
	Heat of reaction, kJ Integrated OTR, 120 min MA	359	2,286	−84%
	Heat of combustion, kJ Consumed methanol	324	2,468	−87%
	*q*_oxygen_, mmol·g^−1^·hr^−1^	0.239	1.235	−81%
	*q*_methanol_, mg·g^−1^·hr^−1^	4.6	37.4	−88%

## 4 DISCUSSION

The Mut ^−^ phenotype of *P. pastoris* has been overlooked in the past years for secreted protein production. There have been attempts to utilize this phenotype by several researchers (Chiruvolu et al., 1997; Inan & Meagher, 2001), but the results were nonconclusive and failed to elucidate the main advantages of the Mut^−^ phenotype. The application scenario of *P. pastoris* today is predominantly for secreted protein production. Experiments previously undertaken on the Mut^−^ phenotype were done with a focus on intracellularly produced proteins, which are subjected to degradation (Zhang, Liu, & Wu, 2007), and thus failed to show the potential of these strains for secreted protein production.

The previously proposed screening method for the Mut^−^ strains is based on using carbon sources such as sorbitol that are not repressing the *AOX1* promoter with up to 0.5% (vol/vol) methanol (Inan & Meagher, 2001). We tested a different approach focused on screening secreted proteins using a slow glucose release system. By cultivating the clones in a glucose limit, the P_*AOX1*_ is first derepressed and later induced by methanol. Longer incubation times and repeated addition of 1% (vol/vol) methanol is necessary (Two shot – extended protocol) to achieve acceptable yields and protein titers. Although the *q*_methanol_ of the Mut^−^ is reduced compared with the Mut^S^ strain, we observed that the P_*AOX1*_ needs higher methanol concentrations to achieve sufficient promoter induction. Still, there is a productivity drop that is consistently observed when compared with the Mut^S^ strain. Promoter expression levels are reduced by 30%, screening yields by 47% and 50% for HSA and vHH, respectively, and the *q_P_* in the methanol only phase by 45%. There are at least two possible explanations for this. Either the cells of the Mut^S^ strain were more metabolically active because they could use two carbon sources at once, or the P_*AOX1*_ is not fully induced in the Mut^−^ strain.

HSA production with Mut^+^ or Mut^S^ strains has already been attempted by some researchers, and by selecting for multicopy clones and optimizing the process, they obtained end titers of up to 10 g/L (Mallem et al., 2014; Zhu et al., 2018). As our work is still at a proof of concept stage, we used single copy clones and did not further optimize the process. In Scenario A, we explored the production of secreted HSA in a Mut^−^ strain by constantly feeding a metabolizable carbon source, as suggested (Chiruvolu et al., 1997). Both the Mut^−^ P_*AOX1*_HSA and Mut^S^ P_*AOX1*_HSA strains are supplied with equal amounts of methanol and glucose; however, the two strains respond differently. The Mut^S^ P_*AOX1*_HSA readily consumes the methanol, and therefore, the methanol concentration is not sufficient to fully induce the P_*AOX1*_. Therefore, the strain performs worse than the Mut^−^ P_*AOX1*_HSA, which is still metabolically active due to the glucose feed and has methanol in excess for P_*AOX1*_ induction. Importantly, this highlights the fact that the Mut^−^ behavior under methanol induction cannot be achieved by simply limiting the methanol feed rate of the Mut^S^ strain. Due to the constant glucose feed rate, the specific growth rate was decreasing as the biomass increased throughout the cultivation. The *q_P_* after an initial spike at the beginning decreases as well. Thus, metabolic activity has an influence on the productivity of the Mut^−^ strain, as seen at higher growth rates at the beginning of the glucose feed. However, it is puzzling that the *q_P_* at low growth rates toward the end of the glucose feed is lower than in the methanol only feed phases of Scenarios B and C. The best performing scenario in terms of productivity was Scenario B that allowed for fast biomass accumulation early in the cultivation coupled with high *q_P_*, followed by a methanol only feed phase with a lower *q_P_* but constant high biomass concentrations. The *q_P_* in the glucose‐methanol co‐feed phase was the highest observed for the Mut^−^ P_*AOX1*_HSA, indicating that the feed was not repressing the *AOX1* promoter. In Scenario C, there was, as expected, no production of protein in the glucose feed phase. Nevertheless, the *q_P_* in the methanol only feed phases are similar, indicating that the induction by methanol in the glucose feed phase or lack thereof had no effect on the productivity in the following methanol only feed phases; further demonstrating, that it is possible to use the Mut^−^ for complete separation of the biomass production phase and the product formation phase. This has been attempted before by inducing the *P. pastoris* MC100‐3 Mut^−^ strain with 0.5% (vol/vol) methanol (Chiruvolu et al., 1997). Product formation of intracellular β‐galactosidase (data provided is in β‐galactosidase units per mg cell mass) started after 20 hr postinduction, and this lasted only for 20 hr when the product activity decreased again. The authors concluded that protein production with the Mut^−^ is not possible. In contrast, we observed a steady and constant product accumulation over the 72 hr long methanol only feed phase, while a slow biomass decrease was observed mostly due to dilution of the culture by the methanol feed.

One important feature of the Mut^−^ phenotype is the possibility to completely separate the growth phase and the protein production phase, as shown in Scenario C, or in the case of Scenario B, the phases can overlap for better overall productivity. This can be utilized for prolonged cultivation times with higher final protein titers as well as for continuous production schemes where the producing biomass is retained for longer periods of time in perfusion/filter bioreactors. *P. pastoris*, although not the Mut^−^ phenotype, was previously recognized as a potential production organism in continuous production schemes (Cankorur‐Cetinkaya et al., 2018). More research may be undertaken on the subject of specific productivity in the methanol only feed phase.

The specific methanol uptake rate of the Mut^–^ strain as calculated from Scenario B is 93% lower than the maximal specific methanol uptake rate determined for *P. pastoris* KM71H Mut^S^ of 62 ± 6 mg·g^−1^·hr^−1^ (Dietzsch, Spadiut, & Herwig, 2011). It was postulated that the Mut^−^ cannot metabolize methanol. It was assumed that the methanol concentration decreases due to stripping from the medium by aeration and agitation (Looser et al., 2015). The evaporation control experiment proved this assumption wrong. This is supported by the CO_2_ in the bioreactor exhaust gas during the methanol only feed phase that was from 0.9% to 1.8% depending on the biomass concentration and the calculated carbon balances. The 120 min moving average RQ values range from 0.65 to 0.70 and are close to the theoretical RQ of 0.66 for complete oxidation of methanol. Measured RQ in the batch phase on glycerol was lower than the theoretical RQ of 0.86, matching the fact that part of the carbon in glycerol is partially oxidized to biomass (Tomàs‐Gamisans, Ferrer, & Albiol, 2018) while in the glucose feed, the measured RQ matched with the theoretical RQ of 1.00 as expected. Taken together this data is an indication that some form of methanol oxidation is ongoing at a low rate in this process. In theory, this could sustain the cells in a viable state as the reported nongrowth associated maintenance energy (NGAME) requirements on methanol for *P. pastoris* X‐33 is 0.44 mmol ATP·g^−1^·hr^−1^ (Tomàs‐Gamisans et al., 2018). This corresponds to only 2.81 mg·g^−1^·h^−1^ of methanol. Thus, the Mut^−^ strain still has a 1.6‐fold higher *q*_methanol_ than required for NGAME. This makes the assumption plausible that the Mut^−^ is at nearly zero growth.

By painting a clearer picture of the benefits and drawbacks of the *P. pastoris* Mut^−^ phenotype, we showed that it has high potential in a variety of applications. The main advantages of the Mut^−^ phenotype are the lower heat output, oxygen demand, and methanol depletion rate as well as higher viability. This leads to less intensive cultivations and therefore cost savings and greater flexibility in terms of reactor systems' choice, as for example single‐use systems with limited OTR and heat dissipation capabilities can be used. Production is achieved with seemingly no biomass increase. The work presented here is at a proof of concept stage and the observed disadvantages, namely lower specific productivity of the Mut^−^ strain, can certainly be overcome by modern strain engineering.

## CONFLICT OF INTERESTS

The authors are inventors of a patent application based on the results reported in this publication.

## Supporting information

Supporting information
