# Supplementary material for: Characterization of methanol utilization negative Pichia pastoris for secreted protein production: New cultivation strategies for current and future applications
Source: Biotechnol Bioeng. 2020 Feb 24;117(5):1394–405. doi: 10.1002/bit.27303 (PMC7187134; doi:10.1002/bit.27303)
Supplement: Supplementary file 1 — Supporting information [file BIT-117-1394-s001.PDF]

Supplementary information to

Characterization of methanol utilization negative *Pichia pastoris* for secreted protein production: New cultivation strategies for current and future applications

Domen Zavec, Brigitte Gasser, Diethard Mattanovich

Supplementary Table 1: qPCR primer sequence used for determining the gene copy number.

Primer name	DNA sequence (5' → 3')
pAOX1_UP	GGCATACCGTTTGTCTTGTTTG
pAOX1_LOW	GTTTCCCCATTTGCGTTTCG
PpACT1_Up	CCTGAGGCTTTGTTCCACCCATCT
PpACT1_Low	GGAACATAGTAGTACCACCGGACATAACGA

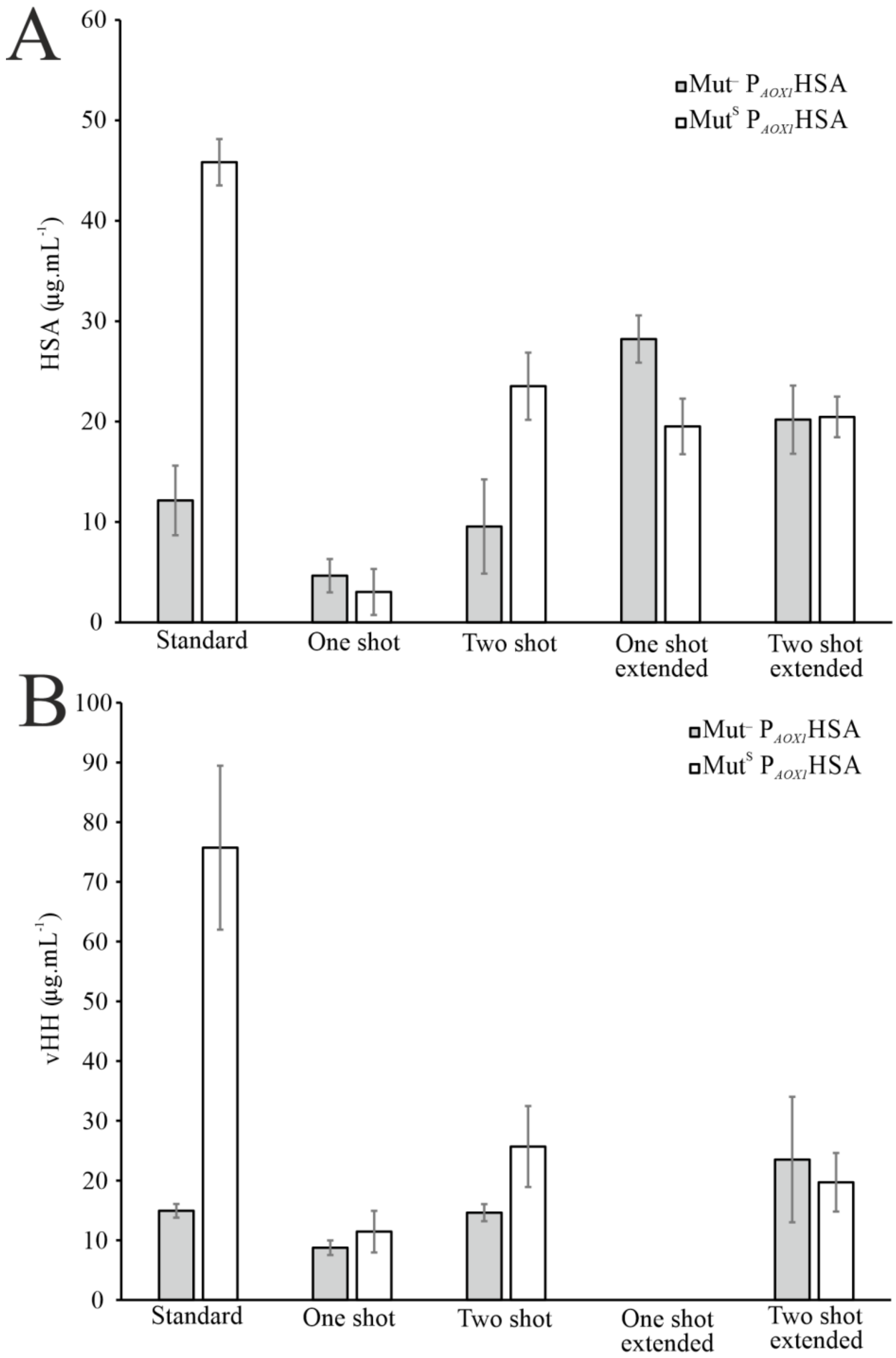

Supplementary Figure 1: Protein concentrations of the small-scale screening.

Supplementary Table 2: Methanol concentration in % (v/v) for all measured time points.

**Scenario A**	**Time (h)**		**43.2**	**68.2**	**92.4**	**116.4**	**120.1**
	Mut$^-$ P_{AOX1}HSA		1.30%	1.19%	1.39%	1.30%	1.31%
	Mut$^-$ P_{AOX1}HSA		1.50%	1.26%	1.35%	1.25%	1.27%
	Mut^S P_{AOX1}HSA		0.0%	0.0%	0.0%	0.0%	0.0%
	Mut^S P_{AOX1}HSA		0.0%	0.0%	0.0%	0.0%	0.0%
**Scenario B**	**Time (h)**	**21.9**	**45.0**	**69.6**	**93.9**	**118.1**	
	Mut$^-$ P_{AOX1}HSA	1.39%	0.90%	1.00%	1.03%	1.02%	
	Mut$^-$ P_{AOX1}HSA	1.44%	0.87%	0.95%	0.89%	1.01%	
**Scenario B**	**Time (h)**	**21.4**	**44.8**	**68.8**	**92.0**	**117.9**	
	Mut$^-$ P_{AOX1}vHH	1.38%	0.99%	1.09%	1.43%	1.33%	
	Mut$^-$ P_{AOX1}vHH	1.37%	1.00%	1.20%	1.24%	1.37%	
**Scenario C**	**Time (h)**		**47.1**	**69.6**	**93.9**	**118.1**	
	Mut$^-$ P_{AOX1}HSA		1.53%	0.54%	0.83%	0.90%	
	Mut$^-$ P_{AOX1}HSA		1.58%	0.75%	0.93%	0.94%	

Supplementary Table 3: Total carbon balance of phase 3, scenario B and C.

Mut$^-$ P_{AOX1}HSA scenario B

Time (h)	Methanol input (gC)	Glucose input (gC)	Total carbon input (gC)	CO_2 Out (gC)	HSA (gC)	Total carbon out & HSA (gC)	Difference
45.0							
69.6	2.08	0.58	2.66	2.53	0.03	2.56	-4%
93.9	1.92	No	1.92	2.04	0.02	2.06	7%
118.1	2.01	No	2.01	1.82	0.01	1.83	-9%
Sum			6.59	6.39	0.06	6.45	-2%

Mut$^-$ P_{AOX1}HSA scenario B

Time (h)	Methanol input (gC)	Glucose input (gC)	Total carbon input (gC)	CO_2 Out (gC)	HSA (gC)	Total carbon out & HSA (gC)	Difference
45.0							
69.6	2.05	0.60	2.65	2.48	0.02	2.51	-5%
93.9	2.00	No	2.00	1.97	0.03	1.99	0%
118.1	1.79	No	1.79	1.75	0.01	1.76	-1%
Sum			6.44	6.20	0.07	6.26	-3%

Mut$^-$ P_{AOX1}HSA scenario C

Time (h)	Methanol input (gC)	Glucose input (gC)	Total carbon input (gC)	CO_2 Out (gC)	HSA (gC)	Total carbon out & HSA (gC)	Difference
45.0							
69.6	1.17	No	1.17	1.38	0.02	1.39	19%
93.9	1.37	No	1.37	1.46	0.02	1.48	8%
118.1	1.44	No	1.44	1.44	0.02	1.46	1%
Sum			3.98	4.27	0.06	4.33	9%

Mut$^-$ P_{AOX1}HSA scenario C

Time (h)	Methanol input (gC)	Glucose input (gC)	Total carbon input (gC)	CO_2 Out (gC)	HSA (gC)	Total carbon out & HSA (gC)	Difference
45.0							
69.6	1.22	No	1.22	1.42	0.02	1.43	18%
93.9	1.44	No	1.44	1.50	0.02	1.53	6%
118.1	1.47	No	1.47	1.47	0.02	1.49	1%
Sum			4.13	4.39	0.06	4.45	8%

Supplementary Table 4: Process parameter overview.

	Scenario A		Scenario B		Scenario C	Scenario D
	Mut^- P_{AOX1}HSA	Mut^S P_{AOX1}HSA	Mut^- P_{AOX1}HSA	Mut^- P_{AOX1}vHH	Mut^- P_{AOX1}HSA	Mut^S P_{AOX1}HSA
Final titer ($mg.L^{-1}$)	384.7	250.4	531.5	1630.9	350.5	910.9
Total protein (mg)	131.3	88.8	174.2	564	104.8	282.0
Final CDW ($g.L^{-1}$)	136.7	129.4	87.8	103.7	94.8	142.2
Overall q_P ($\mu g.g^{-1}.h^{-1}$)	28.6	19.85	45.8	118.0	NA	61.7
Methanol only feed q_P ($\mu g.g^{-1}.h^{-1}$)	NA	NA	34.0	88.2	32.9	61.5
